# E-learning modules to improve clinical reasoning and practice: a prospective comparative study

**DOI:** 10.12688/mep.19449.2

**Published:** 2024-01-05

**Authors:** Fabiola Stollar, Bernard Cerutti, Susanne Aujesky, Daniel Scherly, Mathieu Nendaz, Annick Galetto-Lacour

**Affiliations:** 1 Department of Pediatrics, Gynecology & Obstetrics, Geneva University Hospitals, Geneva, 6 Rue Willy-Donzé, Geneva 1211, Switzerland; 2 Faculty of Medicine, University of Geneva, Geneva, 1 Rue Michel-Servet 1206, Switzerland; 3 Unit for Development and Research in Medical Education (UDREM), Faculty of Medicine, University of Geneva, Geneva, 1 Rue Michel-Servet, 1206, Switzerland; 4 Service of General Internal Medicine, University Hospitals of Geneva, Geneva, 4 Rue Gabrielle-Perret-Gentil, 1205, Switzerland; 5 Pediatric Emergency Division, Children's Hospital,, University Hospitals of Geneva, Geneva, 6 Rue Willy-Donzé, Geneva 1211, Switzerland

**Keywords:** knowledge acquisition, e-learning, traditional lectures, clinical skills in pediatrics, medical education, learning method, student satisfaction

## Abstract

**Background:**

Controversy remains about whether e-learning can improve clinical competences. Our study aimed to compare the effects of e-learning versus traditional education on medical students' reasoning and how they applied their knowledge to clinical competences, assess factors associated with e-learning that might influence exam scores, and evaluate medical students' satisfaction with these two learning methods.

**Methods:**

Prospective study of 299 medical students in two fourth-year pediatric clerkship cohorts (2016–17 and 2017–18) in Switzerland.

**Results:**

We found no evidence of a difference in students' reasoning or how they applied their knowledge to competences in clinical case resolution, whether they had followed e-learning modules or attended traditional lectures. The number of quizzes taken and being female were factors associated with better scores. Even though overall satisfaction with the two learning methods was similar, students claimed that they learned more in e-learning than in traditional lectures and that e-learning explained learning objectives better.

**Conclusions:**

E-learning could be used as a supplement or alternative to traditional face-to-face medical teaching methods without compromising teaching quality. E-learning modules should be better integrated into medical students' curricula but avoid the risk of curriculum overload, especially in case of repeated COVID-like context.

## Introduction

The processes of teaching and learning are domains in constant development. Previous studies have shown that traditional educational models have several limitations, mainly temporal and spatial ^1^. The United Kingdom's General Medical Council has made significant contributions to shaping today's undergraduate medical curriculum, which had been considered overloaded ^2^. For over a decade, medical schools have been working to transform teaching by reducing the number of lectures, implementing team-based and self-directed learning, or promoting individualized, interprofessional education ^3, 4^.

Computers and the internet are used more and more in educational settings. Although e-learning means different things to different people, in its broadest sense, it is the use of the internet for education. E-learning's pedagogical approach typically aspires to being flexible, engaging, and learner-centered. It overcomes students' and teachers' geographical and temporal constraints and encourages collaboration and communication ^5^. Virtual learning programs, available any time, facilitate access to knowledge and add interactivity to study, encouraging reflection and problem solving and stimulating independent learning and self-discipline ^6^.

The emergence of coronavirus disease (COVID-19) significantly disrupted medical education and required the intensive, prompt attention of medical educators. The University of Geneva's Faculty of Medicine quickly transferred the theoretical parts of the curriculum to an online format. Small groups convened online in virtual team settings, and clinical skills sessions either occurred online or were deferred ^7^. Most small group lectures and tutorials had to become live remote courses. This often added to the workload of doctors and faculty already burdened with the large influx of COVID-19 patients ^8– 10^. The present study was carried out before the coronavirus pandemic, but it now has important implications given that the current context is forcing us to adapt teaching formats.

A recent systematic review found that distance education was not different from traditional education in terms of effectiveness ^11^. A Cochrane review also compared e-learning to traditional learning and showed albeit with low certainty of evidence that e-learning made little or no difference to health professionals' skills and knowledge ^12^. However, these reviews did not test competences in clinical case resolution, which is a core competence of medical education, but the outcomes measured were health professionals' knowledge retention and behaviors. To compare these two teaching methods, this study analyzed level 1 of Kikspatrick's model by means of a satisfaction questionnaire sent to students, and level 2 by assessing the success score of questions testing clinical case resolution ^13^.

It thus seemed appropriate to move beyond testing learning equivalence and important to evaluate competences in clinical case resolution, assess the factors associated with better learning, and investigate whether some groups succeed more than others. Furthermore, recent studies suggest that combining face-to-face training with e-learning is more flexible than other educational methods ^14, 15^. The challenge for the sector will be to identify optimal ways of using e-learning as part of a blended approach to learning in clinical settings ^16^.

The present research was carried out to compare the effects of e-learning and traditional lecture-based training on medical students' reasoning and how they applied their knowledge to clinical case resolution by evaluating their performance in a written pediatrics exam. Our secondary aims were to evaluate medical students' satisfaction with these two different learning methods and assess the factors associated with e-learning that might influence exam scores, such as the numer of quizzes taken online, the quiz performance, and gender.

## Methods

### Study design and subjects

This was a prospective study involving two cohorts of medical students in the fourth year of their six-year curriculum and participating in an eight-week pediatric clerkship rotation (2016–17 and 2017–18 intakes) at the University of Geneva's Faculty of Medicine, Switzerland. Three students repeating a grade due to failing on the previous year were excluded. 

### Ethics and consent

The study protocol was submitted to the Cantonal Research Ethics Commission of Geneva and was exempted from a full review process as it does not fall under the Article 2 of the Swiss Federal Law on research on human beings as it involved existing anonymized data, and was designed to evaluate the quality of undergraduate or postgraduate educational programs (authorised by the President of the Committee Dec 22 ^nd^ 2015 via email). According to a 2009 decision made by the Ethics Committee of Geneva and the University of Geneva’s Faculty of Medicine Teaching Committee Office, research projects in the field of medical education, dealing with existing anonymized data, and designed to evaluate the quality of undergraduate or postgraduate educational programs, are exempt from the need for a full review process and formal approval by the Ethics Committee. The participant consent was waived by the committee. All the data were completely de-identified in accordance with the Safe Harbor method. All files and records were stored in local institutional data servers. This study followed the Strengthening the Reporting of Observational Studies in Epidemiology ( STROBE) reporting guideline for observational studies.

## Learning activities

### Traditional learning

Medical students participate in several supervised clinical activities during their pediatrics clerkship and attend a standardized program of traditional case-based tutorials and lectures held for each clerkship rotation.

### Online e-learning course format

Geneva Children's Hospital has set up an educational website that allows medical students to achieve various learning objectives in pediatrics through individual learning using case-based scenarios. These deal with the most common situations faced in general pediatrics, neonatology, pediatric orthopedics, and pediatric surgery. Many of the learning objectives are covered exclusively in e-learning modules available on the educational website, which is available 24/7. All students were well familiar with e-learning because from their baccalaureate they have access to e-learning.

E-learning modules use the Moodle ^TM^ (Moodle Pty Ltd, West Perth WA 6872, Australia) platform. Each learning activity is structured as follows:

- An introduction including statements about specific learning objectives;
- A topic covered using a step-by-step approach, including one or several case-based vignettes; question–answer–feedback sections alternate with theoretical learning content throughout the resolution of the case;
- A quiz section enabling students to review the learning content; key features of the clinical case's diagnosis and management are tested using multiple-choice questions; several attempts per quiz are allowed, and students can review their attempts throughout their clerkship; quiz settings also allow students to see the correct answers, their scores, and get some feedback immediately after their attempt.

## Pediatric exam evaluation method

Students take a written exam once, after they have completed their clerkship: students who did their clerkship between January and April (two groups of 30 students each) take the May exam; those who did their clerkship between June and December (three groups of 30 students each) take the January exam. The latter students thus have a more extensive overall clinical and theoretical background since they have completed clerkships in other fields in the meantime.

**Figure 1.**
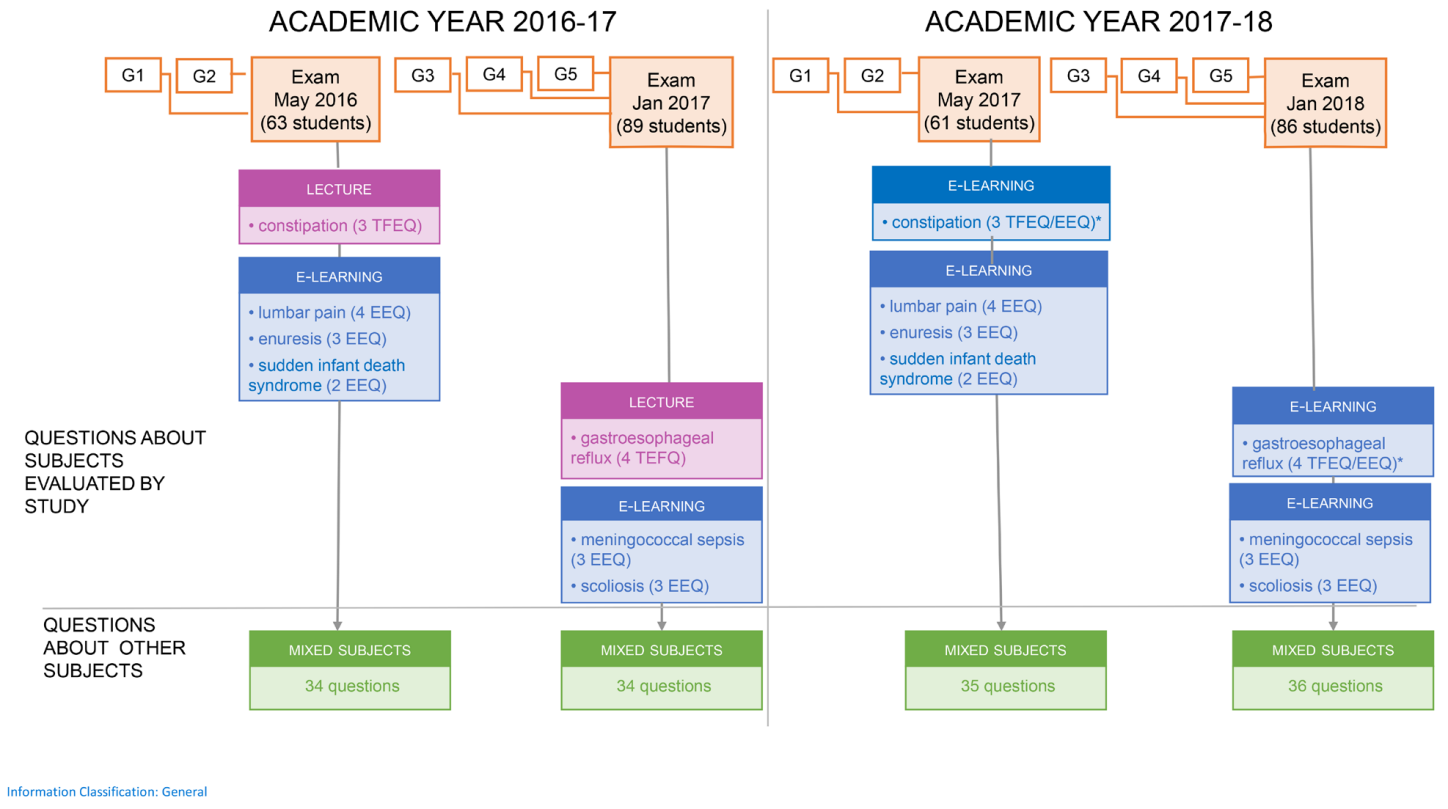
Subjects of questions by teaching format. Definitions: Mixed subjects: Different subjects and different questions used for each exam, these subjects were taught by lectures, Problem Based Learning- vignettes and/or e-learning chapters. Teaching format experimental questions (TFEQ): the computer-based written examination (CBWE) questions related to the constipation and gastroesophageal reflux, the two subjects taught either by traditional lectures in 2016/2017 or by e-learning in 2017/2018. E-learning experimental questions (EEQ): the CBWE questions on the seven subjects taught exclusively by e-learning. All experimental questions were kept identical during both May sessions as well as during both January sessions, to compare students with the same background. * These questions were both analyzed as TFEQ and EEQ.

Exams are online and use either the CAMPUS (for computers) or tEXAM (for tablets) software provided by the Umbrella Consortium for Assessment Networks UCAN, Heidelberg, Germany). Exam content is designed to test clinical reasoning skills and theoretical knowledge. Students must deal with the step-by-step management of several clinical situations presenting different common pediatric complaints. Supplemental patient information, given sequentially, allows them to move towards case resolution ^17^. ( Figure 1)

Clinical situations and question content are based on the learning objectives of the clerkship. Questions are developed by pediatric hospital faculty and reviewed in a multi-step procedure by a panel of experienced pediatric faculty. Before its utilization, each exam is tested on the computer/tablet by several members of the reviewer panel.

## Comparing e-learning and traditional lectures

To compare the e-learning and lecture formats, two e-learning modules (on constipation and gastroesophageal reflux) were taught exclusively using traditional lectures (the 2016–17 academic intake) and exclusively using e-learning modules (the 2017–18 academic intake). Lectures were given by the authors of the e-learning modules so as to maximize similarities between the learning content in the lectures and e-learning modules. Students from the 2016–17 academic intake could not access any parts of the constipation and gastroesophageal reflux e-learning modules (introduction section, case-based vignette, or quiz) ( Figure 1). We named the questions relating to these two subjects the *teaching format experiment questions* (TFEQ) as they tested the two learning formats (e-learning and traditional), and we analyzed and compared the TFEQ scores for these two academic intake groups.

## Factors influencing scores in subjects taught using e-learning

Questions on subjects taught exclusively using e-learning (seven modules; Figure 1) were named the e-learning experiment questions (EEQs). The following factors were considered: exam scores (excluding TFEQs and EEQs), the number of quizzes taken, quiz score, gender, and the cohort effect.

Access to every e-learning module made by individual students using their institutional login was documented. We considered the number of attempts that each individual made for each quiz, as well as the highest quiz score they obtained for each quiz.

Experimental questions ( Figure 1) were asked in the same session of the following year to avoid a "session effect", as we had previously shown in a retrospective study that our students were more likely to get higher scores in exams taken in the second session (January) than in the first (May). This difference could be explained by the fact that medical students taking exams in May have less overall clinical experience ^18^.

### Exam sessions (May exam and January exam)

Each exam session also included 34–36 other items (i.e., non-experimental) covering a range of related topics. These items were different in each exam session. The topics tested using these items were taught in lectures, problem-based learning tutorials, and/or e-learning chapters. Thus, corresponding scores could be used as controlling factors for estimating each student's level.

## Evaluation of student satisfaction

All the students were invited to evaluate teaching activities (on knowledge acquisition, the clarity of learning objectives, achieving learning objectives, curriculum adequacy, teacher preparedness, e-learning, and traditional lecture or tutorial learning activities) at the end of their eight-week pediatrics clerkship. Four-point Likert scale items were used in all our institutional surveys. We also analyzed the free text comments left in the questionnaires, without the use of assistive software.

## Analysis

Scores (0 to 100) were given as the percentage of points scored divided by the maximum points achievable. Data were summarized using mean, median, and inter-quartile range (IQR), and Student's t-tests or Kruskal–Wallis rank sum tests were used to compare the two different groups' scores.

We used multivariate linear regressions to investigate associations between EEQ scores and all the exam's other item scores, numbers of quizzes taken, mean scores obtained for the different quizzes (if a quiz was attempted several times, we considered the highest score), gender, and a cohort effect. All the analyses were made using R software, version 4.1.1 (R Foundation for Statistical Computing, Vienna, Austria).

## Results

### Study group

The study group included 299 (43.5% men; 56.5% women) fourth-year medical students from the 2016–17 and 2017–18 academic intakes; median age on the exam date was 24.1 years old (IQR 23.4–24.9). The average exam score was 77.0 (± standard deviation 8.8) for the 2016-17 academic intakes, and 79.7 (± 8.8) for the 2017-18 academic intakes; when excluding TFEQs and EEQs the average exam score was 76.0 (± standard deviation 9.4) for the 2016-17 academic intakes, and 79.6 (± 8.3) for the 2017-18 academic intakes. Regarding the topics always addressed with e-learning (lumbar pain, enuresis, sudden infant death syndrome, meningococcal meningitis, scoliosis) the number of quizzes taken was 2.0 (±2.8) for the 2016-17 academic intakes, and 2.8 (± 3.2) for the 2017-18 academic intakes.

### Comparing e-learning with traditional lectures

**Figure 2.**
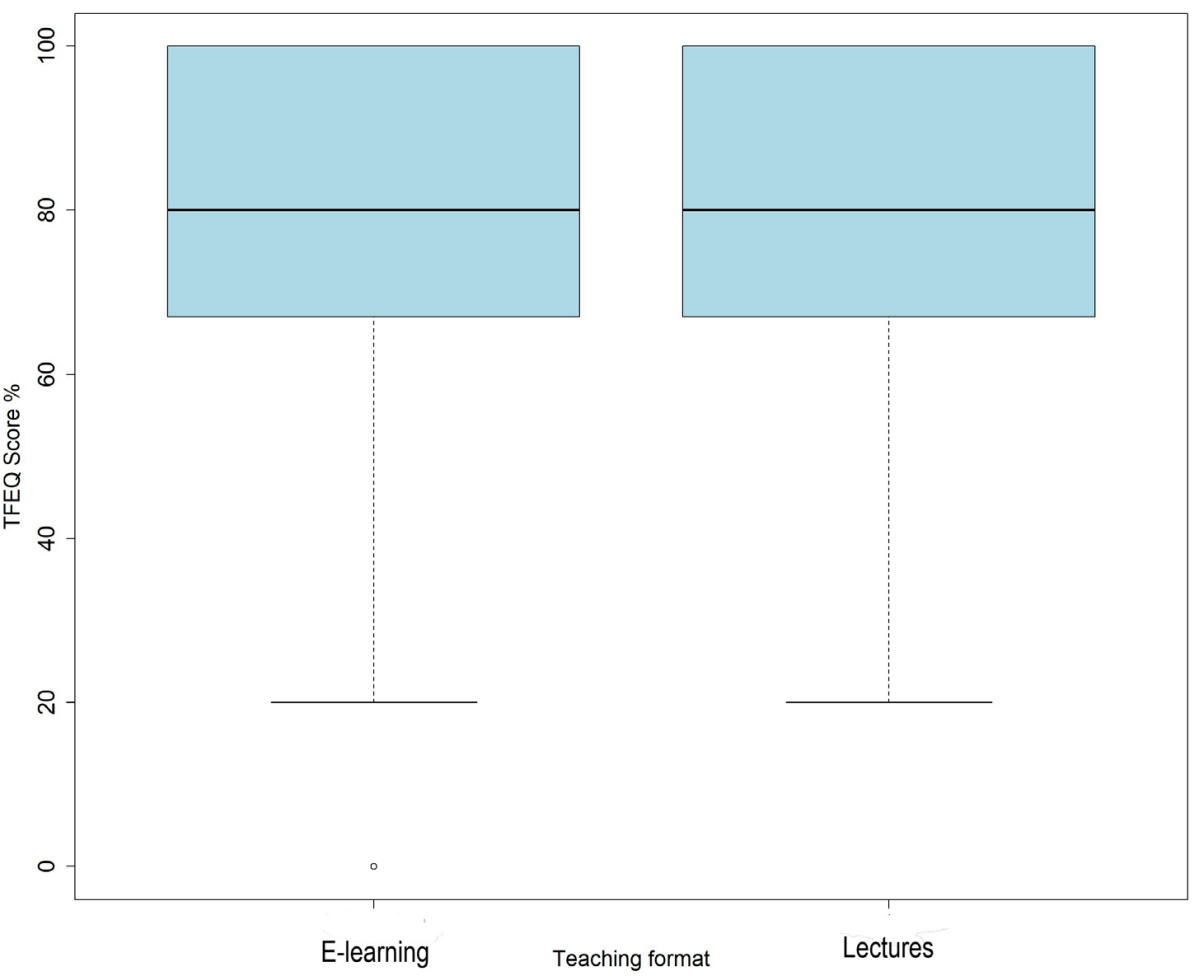
Total combined scores from tests on gastroesophageal reflux and constipation by teaching format. Teaching format experimental questions (TFEQ).

Students exposed to interactive e-learning modules had almost the same TFEQ scores as students exposed to traditional lectures (mean 79%, median 80%, IQR 67%–100% vs. mean 80.3%, median 80%, IQR 67%–100%; *p* = 0.255) ( Figure 2).

### Factors influencing exam scores in subject areas taught using e-learning

A linear regression model showed that EEQ scores were associated with both the number of quizzes taken and gender, irrespective of the scores on non-experiment items ( Table 1).

**Table 1.** Linear regression model of exam scores for items associated with e-learning subjects (EEQ) (R ^2^ = 0.1814).

	Univariate analysis			Multivariate analysis °°		
Covariate	Coefficient	Standard error	*p*	Coefficient	Standard error	*p*
Control score (non-experiment items) *	0.564	0.113	< 0.0001	0.568	0.114	< 0.0001
Number of quizzes taken **	0.715	0.289	0.0142	0.717	0.289	0.0140
Quiz score ***	3.029	1.788	0.0921	-0.213	1.778	0.9048
Cohort effect °	-0.120	2.026	0.9530	-2.691	1.927	0.1645
Gender §	-3.735	2.017	0.0658	-4.140	1.880	0.0290

* range 49–99 (score) ** range 1–18 *** range 2.4–6.0 (points) § Men compared with women. ° Second cohort (2016–17 academic intake) compared with first cohort (2017–18 academic intake). °° Fisher statistic: 7.492 on 5 and 169 degrees of freedom (p <0.0001), R ^2^ = 0.1814

### Survey regarding the two learning methods

Table 2 compares students' evaluation questionnaire responses regarding e-learning and traditional lectures. Even though overall evaluations were almost identical, students reported learning more during e-learning modules than in traditional lectures and that their learning objectives were better explained in the e-learning modules. They felt, however, that traditional lectures were better integrated into the curriculum. Some examples of the positive comments written in the free text sections of the evaluation questionnaire included:

- Web site quality and organization: "Great website. Perfect for learning"; "The website is a real source for studying"; "The learning information is present and easily accessible"; and "Super site, very complete, and very educational".
- Quality of e-learning: "E-learning is great!"; "The complementary e-learning site is a positive point".

However, some examples of the negative comments are:

- Size of the website/e-learning: "Website is very long"; "A complete website, but difficult to approach"; "Too much e-learning, lots of details, not clear what you *need* to know".
- Lack of time for e-learning: "Given the amount of e-learning we are asked to do, there is not enough time planned for it"; "There's not enough time for e-learning".
- Poor integration of e-learning: "Lectures and e-learning scenarios sometimes overlap. It is not easy to get an overview".

**Table 2.** Satisfaction questionnaire comparing e-learning and traditional lectures (N = 201 respondents) *.

	E-learning	Traditional lectures	
	Mean ± SD	Mean ± SD	*P*
**I learned a lot during this activity**	**3.70 ± 0.46**	**3.37 ± 0.56**	**< 0.0001**
**The learning objectives are clearly explained**	**3.34 ± 0.73**	**3.21 ± 0.65**	**0.0259**
I was able to achieve the learning goals	3.22 ± 0.61	3.30 ± 0.57	0.2166
**The activity is well integrated into the curriculum**	**3.16 ± 0.79**	**3.40 ± 0.59**	**0.0002**
The teachers are well prepared	3.54 ± 0.62	3.65 ± 0.48	0.0522
Overall mean score	3.38 ± 0.49	3.38 ± 0.48	0.69074
SD = standard deviation			

*Data could not be desagregated by gender since the teaching evaluation forms used by the institution do not include a Gender item.

## Discussion

### Summary of principal findings

The present study provided evidence to suggest that online learning is at least as effective as traditional teaching formats. Regarding the factors associated with better learning, as expected, exam scores for the items associated with e-learning subjects depended on students' intrinsic capacities and skills. This was illustrated by the fact that the TFEQ and EEQ scores were associated with the scores for non-experimental items. The number of quizzes taken and being a woman student were factors associated with better scores.

### Equivalence between e-learning and traditional learning

We found no differences in the acquisition of reasoning competencies when comparing undergraduate e-learning with traditional lecture-based educational methods. Two recent reviews comparing e-learning with traditional learning also found them to be similarly effective, but they evaluated a different population of learners that was a mix of health professionals, nurses, doctors, and pediatric health consultants, and the Cochrane review even excluded trials involving undergraduate health professionals ^11, 12^.

Another strength of our study was that we compared the two teaching formats through clinical case resolution competences, whereas the existing reviews had only assessed simple knowledge retention and acquisition ^11, 12^. We wanted to test e-learning in a real-life context, with modules integrated into the curriculum to provide a more flexible form of self-learning; one where the students chose the most appropriate moment to study. The format’s impact was tested during end-of-semester exams. This was in contrast to He *et al*., whose systematic review and meta-analysis only included studies using synchronous distance education ( *i.e.*, studies that tested knowledge retention for every learner in a synchronized manner, just after the teaching periods ^11^.

### Beyond testing learning equivalence—factors influencing exam scores

Our study moved beyond testing learning equivalence and evaluated the factors associated with better learning and whether some groups succeed more than others experiencing different teaching methods. The positive impact (better scores) of completing more quizzes was one interesting result. One study evaluating factors influencing examination scores among preclinical medical students showed that students who were highly motivated to study medicine had higher scores ^19^. Those who spent more daily hours preparing for exams, using the internet for academic purposes, and achieving their study targets also ranked higher in the 'excellent' group (scores ≥ 80%) ^19^. Moreover, several publications supported by neuroscience have shown that giving students frequent tests as part of their training is an effective learning method ^20^.

Our study also found evidence that woman students got higher EEQ scores, even when controlled for the student's intrinsic quality and the number of quizzes taken. Men and women show differences in examination performance across the spectrum of medical education, with woman medical students consistently outperforming their men counterparts ^21– 23^, and this trend continues into some postgraduate specialties. Woman doctors are known to perform better in pediatrics, as tested in our study ^23, 24^, and in sex-specific domains like obstetrics and gynecology ^25, 26^. Pope *et al*. also showed that woman trainee general practitioners outperformed their men peers in overall clinical skills assessment, in each individual assessment domain, and in every area of the curriculum. The difference in performance was most marked in the areas of women's health and sexual health and least marked in cardiovascular health, rheumatology, and musculoskeletal health ^27^. Some differences have also been found regarding the use of certain Moodle resources, men's perceptions that e-learning activities interfere with their social life, and women's greater sense of duty ^28^.

## Student satisfaction and evaluation questionnaire

Our study participants gave traditional learning and e-learning similar overall evaluations, and students were satisfied with both learning methods. Liyun He *et al*., however, found that the pooled effect size of overall satisfaction significantly favored e-learning over traditional education ^11^.

It is, nevertheless, important to highlight that our medical students subjectively felt that they learned more during e-learning than during traditional lectures. This may be because e-learning modules repeated important information several times, initially in the specific subject's question format, then in the theoretical explanations following the question, and then in the module's associated quiz. Schiekirka S *et al.* in a systematic review of the factors influencing students' ratings in undergraduate medical education course evaluations indicated that overall course ratings were mainly influenced by students' satisfaction with teaching and exam difficulty, rather than the objective standards of high-quality teaching ^29^. Furthermore, our medical students claimed that they lacked time for e-learning. They may have thought that traditional lectures were better integrated into the curriculum because they occurred at set times in set places, whereas, with e-learning, students had to decide when to study by themselves.

### Study limitations

Our study had some limitations. It was carried out in a single faculty and the results might not be generalizable to others. We included all the students, however, hence avoiding the selection bias of studies conducted among volunteers. We did not evaluate costs or other economic aspects linked to the two teaching methods, but this was beyond the study's scope. We could not evaluate the time students spent online accessing modules because their access was discontinuous: interruptions cannot easily be measured, preventing a reliable quantification of time spent online. We did not pre-assess students' baseline knowledge and how it had developed. Finally, the number of experimental items was small. Further research could examine the issue of students' feelings of isolation during e-learning and its consequences, which this study was unable to do.

Since there were no differences in the success rates for the items used in both sets of exams, this suggests that there was no transmission of information about the questions asked between the 2016–17 intake students and the 2017–18 intake students. However, we cannot be sure that there was no communication between the two groups.

Lastly, it would have been interesting to evaluate clinical reasoning development as a measured outcome of specific teaching strategies using more authentic assessment design principles, but it was beyond the scope of our study specially because we evaluated pre-graduate students, it is too early in their journey to evaluate certain clinical aspects and reasoning

### Implications for future practice and research

The COVID-19 pandemic has caused unprecedented disruption to medical education and healthcare systems worldwide ^30^. This context led us to start discussing potential new ways of replacing traditional learning that would have minimal effects on the progression of students' overall medical training. Transforming tutorials and lectures into e-learning modules could be a long-term solution for freeing up tutors' and students' time without losing quality. However, knowing that this would lead to fewer contacts and interactions between students, it might be wise to encourage them to follow their e-learning modules in groups so as to enhance exchanges between them, boost discussions about clinical case resolution, and mitigate their worries about the e-learning model.

Our study illustrated the importance of completing quizzes that put students into situations that mimic real-life practice using case-based scenarios. It also showed the importance of giving students the opportunity to self-assess after completing a stand-alone e-module. During interactive face-to-face courses, tutors give constant feedback based on discussions with students and the repetition of key points. During an e-learning module done alone, an end-of-module quiz in some way mimics the same role (repeating key points and enabling students to check whether they have understood them). It seems important that e-learning modules be designed so that a clinical case resolution quiz truly contributes to improve students’ clinical competencies. This involves students creating an intellectual process based on theoretical clinical skills and knowledge—training for later implementation in their medical practice.

Timetables should, nevertheless, reserve dedicated time slots for autonomous learning. Traditionally taught and timetabled activities should be linked to e-learning modules so that the latter are better integrated into the curriculum.

Another interesting feature of e-learning is the potential to externalize learning activities. Teaching and learning could be shared with other centers, universities, and hospitals: the technology for more flexible solutions is readily available. Further research should evaluate how to integrate e-learning with traditional methods to avoid curriculum overload and a lack of time for autonomous learning. It could also evaluate differences in examination performance amongst groups of trainees studying different medical specialties, how those differences affect planned educational activities so they can be adapted to students' particular needs, and the new opportunities e-learning offers for learning and teaching.

## Conclusions

We found no evidence of differences in medical students' reasoning or how they applied their knowledge to competences in clinical case resolution between cohorts using e-learning and traditional lecture-based methods. E-learning could be used as a supplement or an alternative to traditional face-to-face medical teaching methods without compromising teaching quality. The number of quizzes taken, and being a woman were the two factors associated with better exam scores, highlighting the importance of self-assessment. Students were satisfied with both learning methods, but they claimed that they learned more through e-learning. We should find better ways to integrate e-learning modules into the medical student curriculum, paying special attention to avoiding curriculum overload, especially if there were to be a repeat of the COVID-19 context.

## Data Availability

Repository: **E-learning modules to improve clinical reasoning and practice** https://doi.org/10.6084/m9.figshare.21769973 This project contains the following underlying data: FIGSHAREPaedeatricELearningData.csv (Dataset regarding the e-learning quizzes and exam socres of the students) FIGSHAREPaedeatricELearningDataREADME.txt (Description of the variables of the dataset regarding the e-learning quizzes and exam socres of the students) FIGSHAREPaedeatricEvaluationSurveyData.csv (Dataset regarding the evaluation of the teaching activities by the students FIGSHAREPaedeatricEvaluationSurveyDataREADME.txt (Description of the variables of the dataset regarding the evaluation of the teaching activities by the students) This project contains the following extended data: FIGSHAREPaediatricNonExperimentalTopicsDuringExams (Exam topics of non experimental questions.) Data are available under the terms of the **Attribution 4.0 International (CC BY 4.0)**
