# Supplementary material for: E-learning modules to improve clinical reasoning and practice: a prospective comparative study
Source: MedEdPublish (2016). 2024 Jan 5;13:39. Originally published 2023 Jul 19. [Version 2] doi: 10.12688/mep.19449.2 (PMC11134138; doi:10.12688/mep.19449.2)
Supplement: Peer review history

# Reviewer report: Reviewer response for version 2

**DOI:** 10.21956/mep.21469.r35769

**Published:** 2024-06-11

**Authors:** Michal Tombs

**Affiliations:** 1 Cardiff University, Cardiff, UK

Many thanks for providing such an extensive response and for making and highlighting all the necessary changes.

Have any limitations of the research been acknowledged?

Yes

Is the work clearly and accurately presented and does it cite the current literature?

Partly

If applicable, is the statistical analysis and its interpretation appropriate?

Yes

Are all the source data underlying the results available to ensure full reproducibility?

Partly

Is the study design appropriate and is the work technically sound?

Yes

Are the conclusions drawn adequately supported by the results?

Yes

Are sufficient details of methods and analysis provided to allow replication by others?

Yes

Reviewer Expertise:

Evaluation

I confirm that I have read this submission and believe that I have an appropriate level of expertise to confirm that it is of an acceptable scientific standard.

# Reviewer report: Reviewer response for version 2

**DOI:** 10.21956/mep.21469.r36846

**Published:** 2024-05-28

**Authors:** Esin Ergonul

**Affiliations:** 1 Department of Medical Education, Dokuz Eylul Universitesi, Izmir, İzmir, Turkey

**Article summary**

 The article 'E-learning modules to improve clinical reasoning and practice: a prospective comparative study', by Fabiola Stollar et al, investigates the effectiveness of e-learning compared to traditional face-to-face teaching in improving medical students' clinical reasoning skills. The study, which was conducted over two academic years (2016-17 and 2017-18), involved 299 fourth-year medical students. The primary aim of the study was to assess the impact of an e-learning module on students' application of knowledge and reasoning in the clinical setting. Secondary aims included assessing student satisfaction with the two learning methods and identifying factors influencing exam scores.

 The study concluded that there were no significant differences in clinical reasoning skills between students who used e-learning and those who attended traditional lectures. However, the number of quizzes taken and gender (female) were associated with higher scores. Students expressed satisfaction with both methods, but many reported that they felt they learned more from the e-learning modules.

**Detailed checking**

**Is the work clearly and accurately presented and does it cite the current literature?**

 Yes: The paper is well written, clearly presented and extensively referenced to relevant and current literature. It provides a thorough review of existing studies and places the research within the broader context of medical education.

**Is the study design appropriate and is the paper technically sound?:**

 Partially:  The study design is generally appropriate, but there are areas for improvement:

**Conceptual framework:** While the revised version introduces the Kirkpatrick model, this model should be more fully integrated into the design and discussion of the study to provide a clearer theoretical basis.

**Methodological details: **More information is needed on the validity and reliability of the all assessment tools. 

**Are sufficient details of methods and analyses provided to allow replication by others?**

 Yes

**Is the statistical analysis and its interpretation adequate?**

 Yes

**Are any limitations of the research acknowledged?**

 Yes: The article acknowledges several limitations, including the single-faculty study setting, potential selection bias, lack of pre-assessment of baseline knowledge, and the small number of experimental items. It also mentions the difficulty of accurately measuring students' online time and the lack of authentic assessments of clinical reasoning.

**Are all source data underlying the results available to ensure full reproducibility?**

 Yes.

**Are the conclusions drawn adequately supported by the results?**

 Yes: The conclusions are well supported by the results. The study finds no significant differences in reasoning skills between e-learning and traditional methods, which is consistent with the data presented.

Have any limitations of the research been acknowledged?

Yes

Is the work clearly and accurately presented and does it cite the current literature?

Yes

If applicable, is the statistical analysis and its interpretation appropriate?

Yes

Are all the source data underlying the results available to ensure full reproducibility?

Yes

Is the study design appropriate and is the work technically sound?

Yes

Are the conclusions drawn adequately supported by the results?

Yes

Are sufficient details of methods and analysis provided to allow replication by others?

Yes

Reviewer Expertise:

Medical Education, Public Health

I confirm that I have read this submission and believe that I have an appropriate level of expertise to confirm that it is of an acceptable scientific standard.

# Reviewer report: Reviewer response for version 2

**DOI:** 10.21956/mep.21469.r36847

**Published:** 2024-05-20

**Authors:** Silvia Lizett Olivares Olivares

**Affiliations:** 1 Tecnologico de Monterrey, Monterrey, Nuevo Leon, Mexico

The article compares learning results from different delivery methods: online vs. face-to-face. The information is clear and complete regarding the test and satisfaction questionnaire. 

 The content of the article has been discussed for many years in the field of education. The word practice in the title is misleading. I was expecting to have distance clinical practices with patients. In the section  "Learning activities, Traditional learning" does include supervised actual clinical activities. However, during the online e-learning course, the program only includes hypothetical case-based scenarios.

 I recommend clarifying how the online e-learning course included practices as the title describes. 

Have any limitations of the research been acknowledged?

Yes

Is the work clearly and accurately presented and does it cite the current literature?

Yes

If applicable, is the statistical analysis and its interpretation appropriate?

I cannot comment. A qualified statistician is required.

Are all the source data underlying the results available to ensure full reproducibility?

No source data required

Is the study design appropriate and is the work technically sound?

Yes

Are the conclusions drawn adequately supported by the results?

Yes

Are sufficient details of methods and analysis provided to allow replication by others?

Partly

Reviewer Expertise:

Medical education

I confirm that I have read this submission and believe that I have an appropriate level of expertise to confirm that it is of an acceptable scientific standard, however I have significant reservations, as outlined above.

# Reviewer report: Reviewer response for version 1

**DOI:** 10.21956/mep.20839.r34812

**Published:** 2023-10-05

**Authors:** Michal Tombs

**Affiliations:** 1 Cardiff University, Cardiff, UK

The manuscript presents a comparative study between two teaching methods on clinical reasoning. The context in which it was conducted is well described, and the rationale for conducting the study was presented. This was in essence a quasi-experimental design, where the researchers compared exam performance and evaluation data (satisfaction) between two cohorts in two different years of study. The sample size was appropriate and the analyses accurate. However, certain aspects of the manuscript can be better improved.

 To begin with, clarity and accuracy of the work can be developed further and this can be achieved by placing the study within a conceptual framework. To this end, it is important to provide a synthesis of the literature on the topic and clearly articulate in what way the research has the potential to add to the body of knowledge and to educational practice. A research question should also be provided and this should be driven by the literature and the identification of a problem or research gap / need. What is the evaluation framework that guided the study? e.g., Kirkpatrick? 

 In terms of methodology, more information on the evaluation instrument would have been useful, how it was developed and by whom. This will then provide opportunity for criticality in the discussion and the limitations of such evaluation methods. 

 The discussion is quite extensive but the results should not be reported here. Indeed, the reporting of linear regressions should be better reported and the rationale for using this statistical methods should be provided. Regressions are normally used when a prediction is made, but there are no predictions in this study. If the study is explorative in nature, a Pearson correlation would suffice, as no predictions were made regarding the direction or strength of the relationship between variables. 

 In summary, this study has the potential to contribute to the body of literature, but the manuscript requires some revisions.

Have any limitations of the research been acknowledged?

Yes

Is the work clearly and accurately presented and does it cite the current literature?

Partly

If applicable, is the statistical analysis and its interpretation appropriate?

Yes

Are all the source data underlying the results available to ensure full reproducibility?

Partly

Is the study design appropriate and is the work technically sound?

Yes

Are the conclusions drawn adequately supported by the results?

Yes

Are sufficient details of methods and analysis provided to allow replication by others?

Yes

Reviewer Expertise:

Evaluation

I confirm that I have read this submission and believe that I have an appropriate level of expertise to confirm that it is of an acceptable scientific standard, however I have significant reservations, as outlined above.

# Response

**Published:** 2023-11-23

**Authors:** Fabiola Stollar

**Affiliations:** University Hospitals of Geneva, Switzerland

Please find below all the responses, point by point, regarding the different comments that you have made.  We also attach a copy of the manuscript revised version in which all the changes have been highlighted (track changes). Yours sincerely, Fabiola Stollar and the co-authors

- “The discussion is quite extensive, but the results should not be reported here. Indeed, the reporting of linear regressions should be better reported and the rationale for using these statistical methods should be provided. Regressions are normally used when a prediction is made, but there are no predictions in this study. If the study is explorative in nature, a Pearson correlation would suffice, as no predictions were made regarding the direction or strength of the relationship between variables.” 

Response: The estimated coefficients (included the corresponding estimated variabilities) and associated significance levels are reported in Table 1. For better exhaustivity we added the R ^2^ coefficient, and the Fisher statistic associated with the multiple regression in the caption of Table 1. Pearson correlation and the linear regression both rely exactly on the same underlying linear model. Computing a regression coefficient is nothing else but investigating the linear association between the two variables. Thank you for pointing out that the main objective of the study was not to do make predictions. We have modified the manuscript and replace “predictor” by “associated factor”.  

- “In terms of methodology, more information on the evaluation instrument would have been useful, how it was developed and by whom. This will then provide opportunity for criticality in the discussion and the limitations of such evaluation methods. 

Response: thank you for your comment. We completed the information required in the Methods section (page: 7).    

- “To begin with, clarity and accuracy of the work can be developed further and this can be achieved by placing the study within a conceptual framework. To this end, it is important to provide a synthesis of the literature on the topic and clearly articulate in what way the research has the potential to add to the body of knowledge and to educational practice. A research question should also be provided and this should be driven by the literature and the identification of a problem or research gap / need. What is the evaluation framework that guided the study? e.g., Kirkpatrick?”

Thank you very much for this very interesting proposal. Kirkpatrick framework concept is indeed appropriate for analyzing the impact of two different teaching methods, which is the main objective of this study. This study analyzed level 1 of Kirkpatrick model through a satisfaction questionnaire sent to students, as well as level 2 through the evaluation of the success score of questions testing clinical case resolution. A description of the framework has been added to the introduction. Summaries of the current literature on the subject, highlighting the problem gap concerning the lack of studies comparing e-learning to traditional learning in the context of case-based competence, which is a core competence of medical education, are now described in the fourth paragraph of the introduction. The main research question was: Is there a difference between e-learning and traditional learning in terms of medical students' reasoning and how they apply their knowledge to clinical case resolution? This question is transcribed in the last paragraph of the introduction (page 5). We would also like to include this reference: Kirkpatrick, D. (1996). Revisiting Kirkpatrick’s four-level-model. Training & Development, 1, 54-57. 

# Reviewer report: Reviewer response for version 1

**DOI:** 10.21956/mep.20839.r34215

**Published:** 2023-08-11

**Authors:** Sarah Hyde

**Affiliations:** 1 School of Rural Medicine, Charles Sturt University, Albury, New South Wales, Australia

This was an interesting paper to read which seemed to show some convincing results about the efficacy of e-learning on the teaching of clinical reasoning. The authors make some valid points about the importance of integrating content and having e-learning modules which are well designed. The method used here included case scenarios in both the traditional component and e-learning modules and the modules, in the limited description provided, meet most of the key design principles of authentic and high quality e-learning. There is a vast literature on teaching clinical reasoning using e-learning but the problem is always with the assessment of this key skill. 

 This research showcases a good study design to further evaluate our knowledge of the usefulness of e-learning as a complementary activity to the standard teaching of clinical reasoning in small groups or at the bedside. More information is needed however to replicate this study and evaluate it's validity. For example, did all of your study cohorts agree or consent to be included in the research? What were the characteristics of each of the cohorts? Whilst steps were taken to ensure equivalent learning experiences by having the same staff provide the lectures as who wrote the content, I am wondering if there was a test effect. For example, could the students who completed the e-learning modules have performed better in the exam by virtue of their familiarity with the case study design used in their modules and experience with the self-assessments? The authors have described using the Mixed Subjects results as a control variable, or factor, but this was not evidenced in the reporting of the results. It would be useful to see some reporting of the differences in the Control score, number of tests taken etc for each cohort, other than in the regression. I am also wondering if there were any differences in the May vs January period, and any differences based on the topic studied. I cannot comment on the efficacy of using regression for this sample size but I am wondering if it was the most appropriate analysis to use. 

 I am also wondering about the possibility of using more authentic assessments in these types of studies. Further mention could be made of the need to evaluate clinical reasoning development as a measured outcome of specific teaching strategies using more authentic assessment design principles. As yet there remains little research in relation to this.

 Overall, this was an interesting read and contributes more knowledge to the area in a broad sense, but more information is needed about the methodology and reporting to enhance this for the reader.

Have any limitations of the research been acknowledged?

Partly

Is the work clearly and accurately presented and does it cite the current literature?

Yes

If applicable, is the statistical analysis and its interpretation appropriate?

I cannot comment. A qualified statistician is required.

Are all the source data underlying the results available to ensure full reproducibility?

Partly

Is the study design appropriate and is the work technically sound?

Partly

Are the conclusions drawn adequately supported by the results?

Yes

Are sufficient details of methods and analysis provided to allow replication by others?

No

Reviewer Expertise:

Problem based learning, medical education, evaluation

I confirm that I have read this submission and believe that I have an appropriate level of expertise to confirm that it is of an acceptable scientific standard, however I have significant reservations, as outlined above.

# Response

**Published:** 2023-11-23

**Authors:** Fabiola Stollar

**Affiliations:** University Hospitals of Geneva, Switzerland

Please find below all the responses, point by point, regarding the different comments that you  have made.  We also attach a copy of the manuscript revised version in which all the changes have been highlighted (track changes). Yours sincerely, Fabiola Stollar and the co-authors  

- “The authors have described using the Mixed Subjects results as a control variable, or factor, but this was not evidenced in the reporting of the results. It would be useful to see some reporting of the differences in the Control score, number of tests taken etc for each cohort, other than in the regression. I am also wondering if there were any differences in the May vs January period, and any differences based on the topic studied. I cannot comment on the efficacy of using regression for this sample size but I am wondering if it was the most appropriate analysis to use.”

  Response: The different covariates used in the analyses were all reported on Table 1. Thank you for highlighting the fact that the basic results (control score, number of tests taken) regarding both groups would be useful for the reader. These results were added at the beginning in the Results section. Globally speaking we have frequently observed a difference between the May and the January scores in our institution. Students taking the exam in January have gone through more clinical rotations and generally perform better, likely because their level of general knowledge and skills is slightly more advanced. The linear regression model is simple (which is particularly important because of the limited among of data in this study), usually well understood, and offers the advantage to allow one to introduce of several covariates (including potential confounding factors).    

- “More information is needed however to replicate this study and evaluate it's validity. For example, did all your study cohorts agree or consent to be included in the research? What were the characteristics of each of the cohorts? Whilst steps were taken to ensure equivalent learning experiences by having the same staff provide the lectures as who wrote the content, I am wondering if there was a test effect. For example, could the students who completed the e-learning modules have performed better in the exam by virtue of their familiarity with the case study design used in their modules and experience with the self-assessments?”

Response: We have already reported in the Methods (page: 6) that "The participant consent was waived by the committee". All students were well familiar with e-learning because from their baccalaureate they have access to e-learning. we added this phrase in the Methods (page: 6)   “I am also wondering about the possibility of using more authentic assessments in these types of studies. Further mention could be made of the need to evaluate clinical reasoning development as a measured outcome of specific teaching strategies using more authentic assessment design principles. As yet there remains little research in relation to this.” Response: thank you for your comment, indeed it would be interested to evaluate it, but it was beyond the scope of our study. We included a phrase in the limitations of the study (page 13) : “it would have been interesting to evaluate clinical reasoning development as a measured outcome of specific teaching strategies using more authentic assessment design principles, but it was beyond the scope of our study specially because we evaluated pre-graduate students.
